# VEXAS Syndrome and Alzheimer’s Disease—Are There Connections?

**DOI:** 10.3390/brainsci15060573

**Published:** 2025-05-26

**Authors:** Aleksandra Sowa, Marta Malicka, Magdalena Biernacka, Jan Aleksander Beszłej, Jerzy Leszek

**Affiliations:** Department of Psychiatry, Wroclaw Medical University, 50-367 Wroclaw, Poland; sowaolaa@gmail.com (A.S.); martam3945@gmail.com (M.M.); jan.beszlej@umw.edu.pl (J.A.B.); jerzy.leszek@umw.edu.pl (J.L.)

**Keywords:** Alzheimer’s disease, VEXAS, inflammation, dementia, neurodegeneration, cognitive decline, UBA1

## Abstract

VEXAS syndrome and Alzheimer’s disease (AD), though distinct in clinical manifestations, share overlapping pathophysiological mechanisms, including systemic inflammation, protein misfolding, and vascular dysfunction. VEXAS syndrome, a rare autoinflammatory disorder characterized by somatic UBA1 mutations, systemic inflammation, and hematologic abnormalities, presents primarily in older males. Meanwhile, AD, the leading cause of dementia, involves progressive neurodegeneration driven by amyloid-beta plaques, tau tangles, and chronic neuroinflammation. This article explores potential connections between the two conditions, focusing on inflammation, neurovascular changes and cellular stress. Systemic inflammation observed in VEXAS syndrome may potentiate neuroinflammatory processes in Alzheimer’s disease (AD), as circulating proinflammatory cytokines have the capacity to cross the blood–brain barrier (BBB), thereby inducing glial activation and promoting neuroinflammation. Additionally, coexisting vascular dysfunctions characteristic of both conditions may synergistically contribute to accelerated cognitive decline. Both conditions involve disruption of the ubiquitin–proteasome system, with UBA1 mutations being specific to VEXAS. Given the established role of UBA1 in maintaining neuronal homeostasis, investigating the overlapping and distinct molecular mechanisms may provide valuable insights into their pathophysiology. The review underscores the need for further research to elucidate these links and improve therapeutic strategies, especially for individuals affected by both disorders.

## 1. What Is VEXAS Syndrome?

VEXAS syndrome, first described in 2020, is a rare adult-onset autoinflammatory condition associated with significant systemic inflammation and hematologic abnormalities [1]. The name of the syndrome is an acronym that consists of the first letters of words describing the main features of the disorder: **V**acuoles observed in hematopoietic precursor cells; mutations in the **E**1 ubiquitin-activating enzyme, its **X**-linked inheritance, **A**utoinflammatory manifestations, and **S**omatic mutation origins. The genetic underpinning of VEXAS syndrome is linked to mutations in the UBA1 gene, located on the X chromosome, which encodes the enzyme responsible for initiating ubiquitin activation. Ubiquitination is a fundamental cellular process involved in protein degradation, and its disruption leads to protein accumulation and immune system dysregulation, manifesting as systemic inflammation.

The clinical presentation of VEXAS syndrome is highly variable, with symptoms commonly including recurrent fevers, skin lesions resembling Sweet syndrome (acute febrile neutrophilic dermatosis), cartilaginous inflammation (chondritis), pulmonary infiltrates, macrocytic anemia, and thrombocytopenia [1]. These symptoms can manifest in a variety of combinations, contributing to diagnostic challenges. Recurrent fevers occur in the majority of cases, with reports indicating that 80–90% of patients present with febrile episodes at the onset [1]. These fevers often occur alongside other inflammatory symptoms such as elevated C-reactive protein and ferritin levels, which are hallmarks of systemic inflammation. In some patients, fever episodes may be intermittent, leading to confusion with other autoinflammatory syndromes [1]. Skin lesions are another frequent manifestation, particularly those resembling Sweet syndrome. These lesions occur in approximately 60–70% of patients and are often erythematous, tender, and sometimes ulcerative. They can be confused with infections or drug reactions, complicating the clinical picture [2]. A subset of patients also develop vasculitic skin eruptions or neutrophilic dermatoses, which further contributes to diagnostic ambiguity [1]. Chondritis affects around 50% of patients, with common sites being the ears, nose, and joints [1]. This can lead to painful, deforming inflammation, particularly in the external ears and nasal cartilage, sometimes resulting in airway obstruction or hearing loss. The presence of cartilage inflammation often suggests relapsing polychondritis, but the concurrent presence of systemic symptoms and vacuoles in bone marrow cells makes VEXAS syndrome more likely [1].

Pulmonary infiltrates are found in approximately 30–40% of patients and often present with shortness of breath and hypoxemia [1]. The pulmonary involvement can sometimes be severe, contributing to a higher risk of morbidity and mortality, particularly in older patients [1].

The study on VEXAS syndrome revealed that hematologic complications are common, with macrocytic anemia present in all patients and lymphopenia in 80%. Thrombocytopenia and neutropenia were more frequent in those who progressed to myelodysplastic syndrome (MDS). Bone marrow analysis showed cytoplasmic vacuoles [3] in myeloid and erythroid precursors with hypercellularity and dysplasia. MDS patients had a favorable prognosis, and none developed leukemia [3]. Thrombotic events, such as venous thromboembolism and stroke, were seen in 10 patients [3]. The study underscores the need for monitoring hematologic progression in VEXAS syndrome [3].

Venous thromboembolic events are a particularly concerning aspect of VEXAS syndrome, seen in up to 40% of affected individuals, and contribute to a higher overall mortality rate [1]. These events, often occurring in conjunction with deep vein thrombosis and systemic inflammation, increase the complexity of patient management.

The disease overwhelmingly affects older males due to its X-linked inheritance, with a median age of onset around 60 years [1]. Although rare, females may be diagnosed, typically when somatic mutations occur in hematopoietic stem cells leading to a mosaic pattern of disease [1]. The severity of symptoms in females tends to be milder, likely due to the presence of a second normal X chromosome, which compensates for the mutated gene in many cases [4].

Bone marrow aspirates are a critical diagnostic tool in VEXAS syndrome, revealing vacuoles in myeloid and erythroid precursor cells in almost all cases. This finding, along with the clinical presentation, confirms the diagnosis [1].

**Figure 1:**
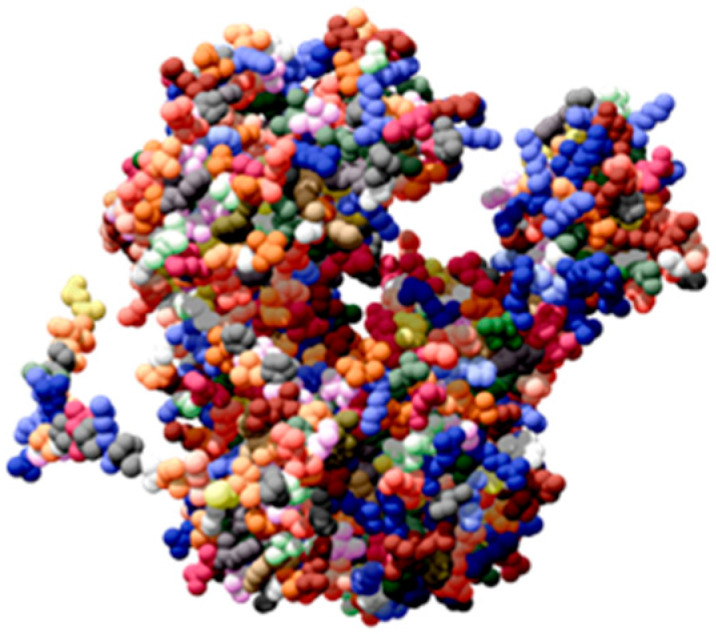
Structure of UBA1. Figure created using AlphaFold (version 2022-11-01) structure predictions (Jumper et al., 2021; Varadi et al., 2024 [6,7]). Different colors represent different amino acids.

Genetic testing for acquired somatic mutations [5] in the UBA1 (Figure 1) gene further supports the diagnosis and can identify somatic mutations in affected patients, thereby providing a definitive diagnostic approach [4]. The vacuolated cells result from defective ubiquitin activation, a key process involved in protein degradation, contributing to the inflammatory and hematologic abnormalities observed in VEXAS syndrome [1].

The diagnosis of VEXAS syndrome requires genetic testing to confirm somatic [8] mutations in the UBA1 gene [9,10]. This testing is essential to distinguish the syndrome from other conditions that mimic it, such as myelodysplastic syndromes or autoimmune disorders [11]. Recent studies employing genome-first approaches have revealed a higher prevalence of VEXAS syndrome than initially anticipated. A population-based study conducted using exome sequencing data from over 160,000 individuals estimated that approximately 1 in 4269 men over the age of 50 carries pathogenic *UBA1* variants associated with VEXAS syndrome, suggesting that the syndrome, while rare, is more widespread than previously understood [12].

**Figure 2:**
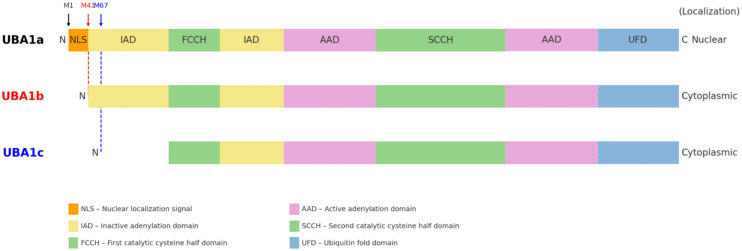
Structure of the UBA1 gene in different isoforms: UBA1a, UBA1b, and UBA1c. N: N-terminus, M1: p.Met1; M41: p.Met41; M67: p.Met67.

The initial reports of VEXAS centered on mutations affecting the methionine-41 codon (p.Met41) of UBA1, but subsequent studies have expanded the mutational spectrum. In addition to the canonical p.Met41 substitutions, at least six novel somatic UBA1 missense mutations—p.His55Tyr, p.Gly477Ala, p.Ala478Ser, p.Asp506Gly, p.Asp506Asn, and p.Ser621Cys—have been identified in VEXAS patients [5]. Molecular characterization of these rarer variants showed that, unlike the Met41 mutations, they do not induce production of the truncated UBA1c isoform; rather, they impair the catalytic function of the normal UBA1 isoforms (UBA1a/UBA1b), highlighting an alternative pathogenic mechanism leading to the same syndrome [5]. The p.Met41 hotspot remains the most prevalent mutation site in VEXAS. In a large cohort from a French registry (*n* = 116), the majority of patients harbored one of the three Met41 variants—approximately 44–49% with p.Met41Thr, 26–30% with p.Met41Val, and 18–19% with p.Met41Leu—whereas splice-site mutations accounted for roughly 5–7% of cases [5,8]. These UBA1^Met41 mutations typically appear as dominant clonal events in hematopoietic cells, with a high variant allele frequency (often ~75% at diagnosis) [13]. This indicates strong positive selection for the Met41-mutant clone in VEXAS patients and aligns with the severe hematologic manifestations observed. Importantly, genotype–phenotype correlations have emerged, linking specific UBA1 variants to clinical outcomes. Patients with the p.Met41Val genotype tend to present with an undifferentiated inflammatory syndrome and have significantly worse survival compared to those with p.Met41Leu or p.Met41Thr [14]. In one multi-center study of 83 cases, p.Met41Val was an independent predictor of higher mortality, whereas the p.Met41Leu variant was associated with a more favorable prognosis [8,14]. Consistently, ear chondritis (relapsing polychondritis of the auricle), a common VEXAS manifestation, was found to correlate with improved survival, while transfusion-dependent anemia portended decreased survival [14]. These observations suggest that certain clinical features and the specific *UBA1* mutation (Figure 2) can influence the disease course: for example, the valine substitution at Met41 confers a higher risk of severe outcomes, whereas the leucine substitution appears more benign in comparison [8,14]. At the molecular level, the three hotspot Met41 mutations all disrupt the normal translation initiation of the cytoplasmic isoform of UBA1 (known as UBA1b). The p.Met41 variants replace the canonical AUG start codon with a non-AUG codon—GUG for p.Met41Val, ACG for p.Met41Thr, and CUG for p.Met41Leu—thereby reducing translation from Met41 and forcing a downstream start at Met67 [14]. This results in the production of an N-terminally truncated enzyme (termed UBA1c) that is catalytically impaired [14]. In vitro studies have demonstrated genotype-specific effects on residual UBA1b production: the p.Met41Val mutation supports substantially less UBA1b translation than either p.Met41Leu or p.Met41Thr [14]. This “cytoplasmic isoform swap”—reduced UBA1b and increased UBA1c—provides a mechanistic explanation for the more severe phenotype of the valine variant, linking lower levels of functional UBA1b to higher mortality in VEXAS [14].

Therapeutic options for VEXAS syndrome remain limited, and management poses a significant challenge. Corticosteroids are commonly employed to control inflammation, but patients frequently develop corticosteroid dependency, and the side effects limit their long-term use. Targeted therapies, including Janus kinase (JAK) inhibitors and interleukin-6 (IL-6) inhibitors, are being explored in clinical trials, showing potential benefits for managing refractory cases [11]. Hematopoietic stem cell transplantation has also been investigated as a curative treatment for select patients, although its risks and long-term outcomes remain areas of ongoing research [2]. Despite these emerging strategies, the prognosis for VEXAS syndrome remains guarded, with significant morbidity and mortality risks driven by its systemic nature and refractory course. VEXAS syndrome underscores the intersection of genetic research and clinical practice, illustrating how somatic mutations can give rise to novel disease entities with profound clinical implications. Advances in understanding its pathophysiology have paved the way for targeted therapies, yet much work remains to optimize diagnosis, management, and outcomes for affected individuals. Continued research is essential to address these challenges and improve the quality of life for patients with this complex and multifaceted condition.

## 2. Alzheimer’s Disease—Brief Reminder

Alzheimer’s disease (AD) is the most common cause of dementia worldwide. Dementia is a condition that affects memory, thinking, language, and problem-solving abilities. According to a 2013 WHO report on the global prevalence of AD, the number of people living with dementia is expected to triple by 2050, from about 35.6 million in 2010. The risk of developing dementia increases with age, affecting around 5–8% of individuals over 65, with the percentage rising to 25–50% for those over 85. The prevalence of AD is 19–29% lower in men than in women [15].

### 2.1. Clinical Features of Alzheimer’s Disease

Alzheimer’s disease primarily affects elderly individuals, though it can also occur in younger populations, in rare cases of early-onset Alzheimer’s (aged 30–60 years). The most notable clinical feature of AD is progressive memory loss, particularly affecting short-term memory, and the gradual inability to recall familiar names or faces [1]. As the disease advances, cognitive functions such as problem-solving, decision-making, and language skills are also impaired, which impacts the individual’s ability to perform everyday tasks independently [16].

In addition to cognitive symptoms, individuals with AD may experience behavioral changes such as aggression, agitation, and depression, which significantly affect both patients and caregivers [17]. The course of the disease is highly variable, but most individuals with AD ultimately require full-time care.

### 2.2. Diagnosis and Current Treatment

Diagnosis of AD is based on clinical criteria, and cognitive tests, imaging studies, and biomarkers are used to confirm the diagnosis. Magnetic resonance imaging (MRI) scans can reveal structural changes in the brain, such as hippocampal atrophy, and can help differentiate it from vascular dementia. Positron emission tomography (PET) scans measure abnormal deposits of a protein called beta-amyloid (Aβ) in the brain and can also detect abnormal accumulation of tau protein. Cerebrospinal fluid (CSF) analysis can detect increased levels of Aβ 42 proteins. The Aβ 42/Aβ 40 ratio and levels of t-tau and phospho-tau (p-tau 181)—the main components of tau tangles in the brain—are also important. Increasingly, tests for these proteins in serum are becoming part of clinical practice [18].

There is currently no effective therapy for AD, and treatment focuses mainly on relieving symptoms and slowing disease progression. Cholinesterase inhibitors (e.g., donepezil, rivastigmine) and glutamate regulators (e.g., memantine) are commonly prescribed to control cognitive symptoms [19]. More recently, monoclonal antibodies directed against beta-amyloid, such as aducanumab, lecanemab, and donanemab, have been introduced into treatment, although their clinical efficacy is limited and effects are achieved only in the early stages of the disease [20].

### 2.3. Neuropathology of Alzheimer’s Disease

The hallmark neuropathological features of AD are extracellular amyloid-beta (Aβ) plaques and intracellular neurofibrillary tangles (NFTs) composed of tau protein. Amyloid plaques are clusters of misfolded Aβ peptides that accumulate between neurons, disrupting cell function and triggering neuroinflammation [21]. On the other hand, tau protein, which normally stabilizes microtubules in neurons, becomes hyperphosphorylated in AD, leading to the formation of tangles inside neurons, further disrupting cellular functions [22].

These pathological changes result in widespread neuronal loss and synaptic dysfunction, particularly in regions of the brain associated with memory and learning, such as the hippocampus and the cerebral cortex [23]. Over time, the progression of neuronal degeneration leads to the profound cognitive impairments characteristic of AD.

## 3. Inflammatory Process and Its Role in Alzheimer’s Disease

Inflammation is a fundamental biological response aimed at repairing tissue damage and defending against pathogens. In the context of AD, this process becomes chronic and contributes to the disease’s progression. Under normal circumstances, inflammation resolves once the injury is repaired. However, in AD, the inflammatory response persists, leading to ongoing neuronal damage. This chronic inflammation is primarily localized in regions of the brain affected by amyloid-beta (Aβ) plaques and tau tangles, two hallmark features of AD pathology.

The inflammatory response in AD is initiated by the deposition of Aβ plaques, which trigger the activation of microglia and astrocytes, the brain’s resident immune cells. These activated glial cells produce a variety of proinflammatory molecules, including cytokines, chemokines, reactive oxygen species (ROS), and reactive nitrogen species (RNS), which exacerbate neuronal damage [24]. Unlike acute inflammation, which is characterized by pain, heat, and swelling, the inflammation in AD is often subclinical and primarily manifests as the activation of immune cells in response to cellular damage [25].

### 3.1. Role of Microglia and Astrocytes in Neuroinflammation

Microglia are the primary immune cells of the CNS, responsible for maintaining homeostasis and responding to brain injury. In AD, microglia become activated in response to Aβ accumulation and tau pathology, contributing to the chronic inflammatory state. These activated microglia release proinflammatory cytokines such as tumor necrosis factor-alpha (TNF-α) and interleukin-1 beta (IL-1β), as well as ROS and RNS, all of which can lead to neuronal damage and synaptic dysfunction [26,27]. Astrocytes, another type of glial cell, also play a significant role in neuroinflammation. Upon activation, astrocytes produce similar inflammatory mediators and contribute to the neurotoxic environment in AD [28].

The interaction between microglia, astrocytes, and neurons creates a vicious cycle of inflammation. Aβ plaques stimulate glial cells to release inflammatory mediators, which, in turn, exacerbate Aβ deposition and tau phosphorylation. This feedback loop accelerates the progression of neurodegeneration and synaptic loss, further impairing cognitive function [29].

### 3.2. Inflammatory Mediators and Their Impact on Neuronal Function

Activated glial cells in AD release a wide range of inflammatory mediators, including cytokines, chemokines, and reactive molecules like ROS and RNS. These molecules not only contribute to neuronal damage directly but also modulate key processes involved in AD pathology, such as amyloid precursor protein (APP) processing and tau hyperphosphorylation. For example, inflammatory cytokines enhance the production of amyloid-beta by promoting the amyloidogenic pathway of APP processing, which results in the generation of the toxic Aβ-42 peptide [30].

Moreover, inflammation in AD leads to the activation of neurotoxic enzymes such as inducible nitric oxide synthase (iNOS) and cyclooxygenase-2 (COX-2). These enzymes produce nitric oxide (NO) and prostaglandins, respectively, both of which are implicated in neuronal injury and synaptic dysfunction [31]. Chronic inflammation not only drives the production of Aβ but also inhibits the formation of protective soluble APP, further exacerbating the neurodegenerative process [32].

### 3.3. The Role of Inflammation in Disease Progression and Potential Therapeutic Implications

The inflammatory response in AD is a double-edged sword: while it serves as a protective mechanism initially, aimed at clearing toxic proteins such as Aβ, chronic inflammation can lead to worsening disease pathology. This paradoxical nature of inflammation suggests that modulating the inflammatory response could hold therapeutic potential for AD. Several therapeutic strategies are being explored to target inflammation in AD, including the use of nonsteroidal anti-inflammatory drugs (NSAIDs), microglial inhibitors, and antibodies targeting amyloid-beta [33].

However, clinical trials using NSAIDs have yielded mixed results, raising questions about the safety and efficacy of inflammation-targeting therapies. More recent approaches, such as the development of monoclonal antibodies against amyloid-beta, have shown promise in reducing Aβ plaques, but their long-term efficacy remains uncertain [20].

## 4. Possible Connections Between VEXAS and Alzheimer’s Disease

**Figure 3:**
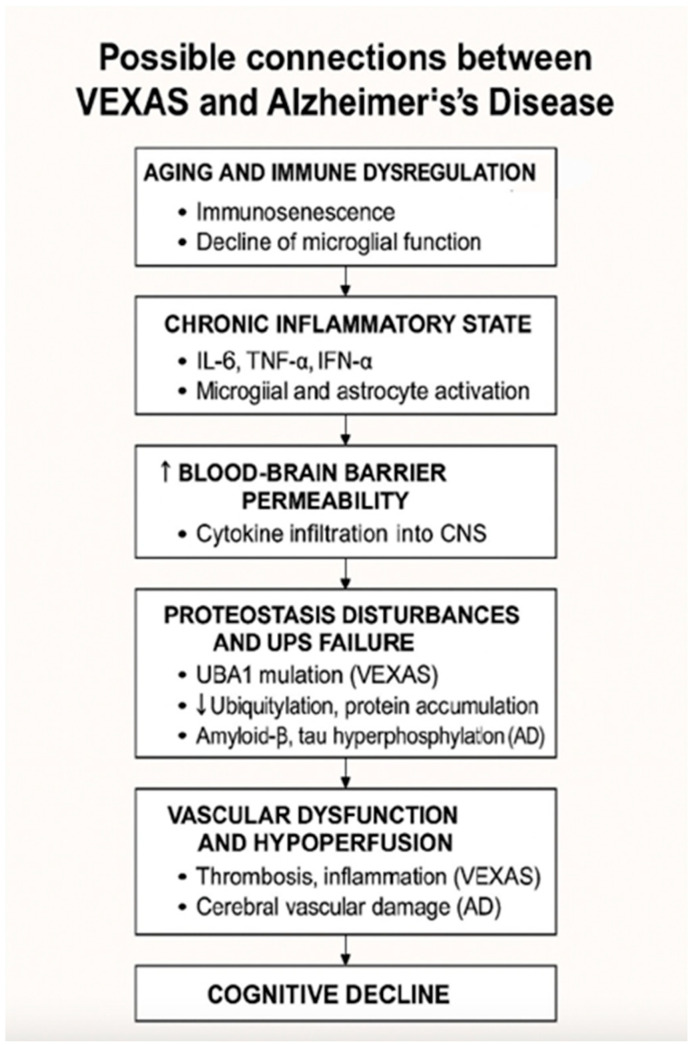
Proposed shared mechanisms linking VEXAS syndrome and Alzheimer’s disease. Immune dysregulation leading to chronic inflammation increase blood-brain barrier permeability, disrupt proteostasis and impair vascular function, ultimately contributing to cognitive decline.

The growing body of research on VEXAS syndrome and AD has brought attention to overlapping pathophysiological mechanisms that may link the two conditions, despite their distinct clinical and genetic presentations. Both diseases share common features such as systemic inflammation, vascular dysfunction, and aging-related immune dysregulation. These shared pathways provide a compelling reason to explore the potential connections between VEXAS syndrome and AD, particularly with respect to inflammation, neurovascular changes, protein misfolding, and cellular stress (Figure 3).

### 4.1. Inflammation and Neuroinflammation

A prominent characteristic of VEXAS syndrome is systemic inflammation, which results from somatic mutations in the UBA1 gene, leading to myeloid and erythroid precursor dysfunction [1]. Patients with VEXAS syndrome experience a chronic inflammatory state that affects multiple organs and may involve features like recurrent fevers, skin lesions, and chondritis. This inflammation, while systemic in VEXAS, bears similarities to the neuroinflammatory response observed in AD, where activated microglia contribute to the pathogenesis of amyloid plaque formation and tau deposition in the brain [4]. In both conditions, an overactive immune system leads to tissue damage, though in AD, this is primarily confined to the central nervous system.

The chronic inflammatory state in VEXAS syndrome may exacerbate neuroinflammation, thus accelerating the development of neurodegenerative conditions like AD. Systemic inflammatory cytokines in VEXAS may be able to cross the blood–brain barrier (BBB), promoting glial activation and neuroinflammation in the brain, similar to what is seen in AD [1]. This shared immunological pathway raises the hypothesis that patients with VEXAS might be at increased risk for cognitive impairment and AD-like neurodegeneration; however, clinical evidence to support this remains lacking, and further research is needed to investigate this potential link.

### 4.2. Vascular Dysfunction and Cognitive Decline

Vascular pathology in both VEXAS syndrome and AD further complicates the clinical picture. In VEXAS, vascular complications such as venous thromboembolism and other forms of vascular inflammation are common. Vascular abnormalities, including small vessel disease and impaired blood–brain barrier integrity, are also central to the pathogenesis of AD, where they contribute to cerebral hypoperfusion and amyloid deposition in the brain [4].

The vascular dysfunction in VEXAS may exacerbate the cognitive decline seen in AD by compromising cerebral blood flow and facilitating the leakage of inflammatory mediators into the brain, further promoting neurodegeneration [11]. This dual vascular burden from both systemic inflammation and local neurovascular damage suggests a potential amplification of cognitive dysfunction in individuals with both conditions, presenting a need for closer monitoring of vascular health in VEXAS patients.

### 4.3. Genetic Mechanisms and Protein Misfolding

**Figure 4:**
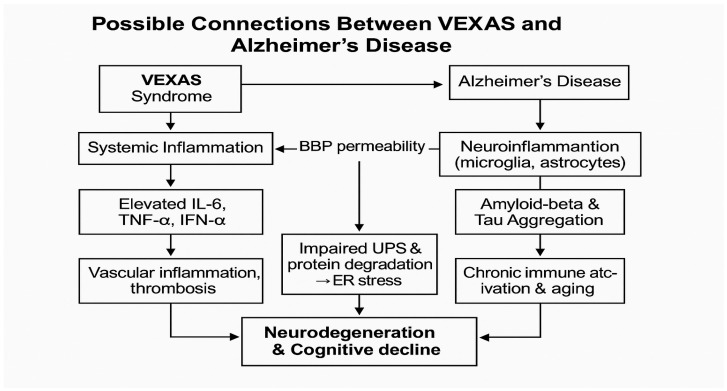
Figure mapping the inflammatory and vascular pathways involved in both disorders.

At a molecular level, both VEXAS syndrome and AD share common cellular stress mechanisms, particularly concerning protein misfolding and accumulation (Figure 4). In VEXAS syndrome, the UBA1 mutation results in dysfunctional protein degradation, leading to the accumulation of misfolded proteins in myeloid and erythroid precursor cells, triggering cellular stress [1]. This accumulation of defective proteins mirrors the amyloid-beta and tau protein aggregation seen in AD, where misfolded proteins cause cellular stress, synaptic dysfunction, and neuronal death.

The ubiquitin–proteasome system (UPS), a central mechanism for maintaining protein homeostasis, appears to be impaired in both diseases. Dysfunction of this system in VEXAS syndrome may contribute to the accumulation of toxic proteins, while in AD, it has been shown to be involved in processes that can promote amyloid plaque formation and tau hyperphosphorylation [15]. These shared stress responses highlight the potential for cross-talk between the mechanisms of protein misfolding in VEXAS syndrome and AD, suggesting that disruptions in the UPS could accelerate neurodegeneration in both conditions. The central molecular defect in VEXAS syndrome is a somatic mutation in the UBA1 gene, most commonly at methionine-41 (p.Met41) in exon 3. [34] This mutation selectively impairs the cytoplasmic isoform of the E1 ubiquitin-activating enzyme, while leaving the nuclear isoform largely intact [1]. The E1 enzyme is essential for initiating the ubiquitin–proteasome system (UPS) by activating ubiquitin and transferring it to E2 conjugating enzymes. Loss of this function leads to a reduction in overall ubiquitylation activity and defective targeting of abnormal proteins for degradation [35]. As a result, misfolded and damaged proteins accumulate intracellularly, inducing endoplasmic reticulum (ER) stress, which activates the unfolded protein response (UPR). This stress response further engages NF-κB and type I interferon signaling pathways, driving a proinflammatory state at the cellular level [36,37]. These molecular abnormalities have been observed particularly in myeloid precursors, contributing to both ineffective hematopoiesis and the systemic autoinflammatory phenotype characteristic of VEXAS syndrome [35]. The persistent inflammatory environment in VEXAS syndrome is driven by markedly elevated levels of proinflammatory cytokines, notably interleukin-6 (IL-6), tumor necrosis factor-alpha (TNF-α), and interferon-alpha (IFN-α) [38,39]. These inflammatory mediators exert widespread immunologic effects and are frequently accompanied by the dysregulated and prolonged activation of type I interferon pathways, producing a highly reactive systemic immune state [26,40]. In aging individuals, this inflammatory burden increases the permeability of the blood–brain barrier (BBB), facilitating the translocation of circulating cytokines and peripheral immune cells into the central nervous system [29,41]. This permeability shift creates a permissive neurovascular environment for subsequent immune cell infiltration and parenchymal inflammation. In Alzheimer’s disease models, even mild peripheral immune activation is sufficient to trigger T-cell migration into brain tissue and promote amyloid-beta accumulation [42]. Within the CNS, immune-sensitized microglia and astrocytes transform into highly reactive glial cells that intensify local inflammation. [43] Stimulated by IL-6, TNF-α, and type I interferons, these glial populations produce excessive amounts of neurotoxic cytokines and reactive oxygen species, while losing their neuroprotective and homeostatic functions [44,45]. This neuroinflammatory profile accelerates synaptic disruption, promotes tau hyperphosphorylation, and enhances amyloidogenic processing, all of which contribute to progressive cognitive decline [29,42]. Type I interferons, which are prominently elevated in VEXAS, are particularly disruptive in the neuronal environment. Chronic interferon signaling impairs microglial clearance of amyloid-beta and induces synaptic pruning while simultaneously driving astrocytic reactivity toward a neurotoxic phenotype [42,46]. Suppressing interferon signaling in murine AD models restores microglial functionality and improves behavioral performance, underscoring its pathogenic role in neurodegeneration [47]. Together, these findings support a mechanistic framework in which the persistent, interferon-saturated immune environment characteristic of VEXAS syndrome fuels a destructive sequence of neuropathological events that closely mirror those seen in Alzheimer’s disease. The chronically elevated levels of inflammatory cytokines, particularly IL-6, TNF-α, and IFN-α, gradually erode the integrity of the blood–brain barrier by weakening tight junctions and rendering the vascular endothelium more permeable and reactive [48]. Once inside the brain, these circulating molecules continuously stimulate glial pattern recognition receptors, triggering prolonged NF-κB and JAK–STAT signaling, which reinforce inflammatory gene expression and lock glial cells into a hyperactive, proinflammatory state [49]. In this persistently inflamed microenvironment, microglia lose their homeostatic and phagocytic capabilities, become metabolically dysregulated, and begin releasing damaging oxidative molecules and excitotoxic cytokines that accelerate amyloid-beta accumulation [50]. Simultaneously, astrocytes become morphologically distorted and functionally reactive, while IL-6 and TNF-α further activate GSK-3β and CDK5, two kinases that drive pathological tau hyperphosphorylation, fibrillar aggregation, and early synaptic collapse [51]. These converging molecular insults—chronic glial overactivation, proteostatic failure, and tau-driven synaptotoxicity—undermine neuronal viability and push forward the cognitive deterioration emblematic of Alzheimer’s disease.

### 4.4. Age-Related Immune Dysfunction and Cognitive Decline

Age-related immune dysfunction is another factor common to both VEXAS syndrome and AD. In VEXAS syndrome, the disease typically manifests in individuals over the age of 50, with the aging immune system playing a significant role in disease progression [1]. Similarly, AD is often characterized by a decline in immune function within the central nervous system, particularly in the form of reduced microglial surveillance, which exacerbates the neuroinflammatory response [4]. As immune dysfunction increases with age in both conditions, the compounded effect of systemic inflammation and neuroinflammation could lead to an accelerated progression of cognitive decline in individuals affected by both VEXAS and AD. Recent clinical observations have expanded the neurological spectrum of VEXAS syndrome. Documented cases include central nervous system vasculitis, encephalopathic features, and cerebrovascular complications, predominantly in older male patients [52]. These evolving manifestations suggest a mechanistic intersection with Alzheimer’s disease (AD), particularly involving chronic immune dysregulation, progressive vascular pathology, and destabilized proteostasis [52,53]. Somatic mutations in the UBA1 gene impair ubiquitin-mediated proteolytic processes. This dysfunction is increasingly associated with tau hyperphosphorylation, synaptic impairment, and neurodegenerative cascades characteristic of AD [53]. To delineate this intersection more precisely, future research should prioritize longitudinal neurocognitive profiling in VEXAS cohorts. This approach should incorporate cerebrospinal fluid or blood-based biomarker assessments, along with advanced neuroimaging modalities such as magnetic resonance imaging (MRI) and positron emission tomography (PET), to evaluate amyloid and tau accumulation [20]. Patient-specific induced pluripotent stem cell (iPSC)-derived neuronal models bearing UBA1 mutations may elucidate the molecular convergence of autoinflammatory and neurodegenerative mechanisms [53]. Clinically, integrated neurologic and hematologic surveillance—particularly in aging male patients—may enable earlier recognition of cognitive vulnerability [52]. Therapeutic trials targeting immune modulation and proteostasis restoration should incorporate cognitive and neuroinflammatory endpoints to appropriately capture overlapping pathophysiological features [52,53].

## 5. Conclusions

**Figure 5:**
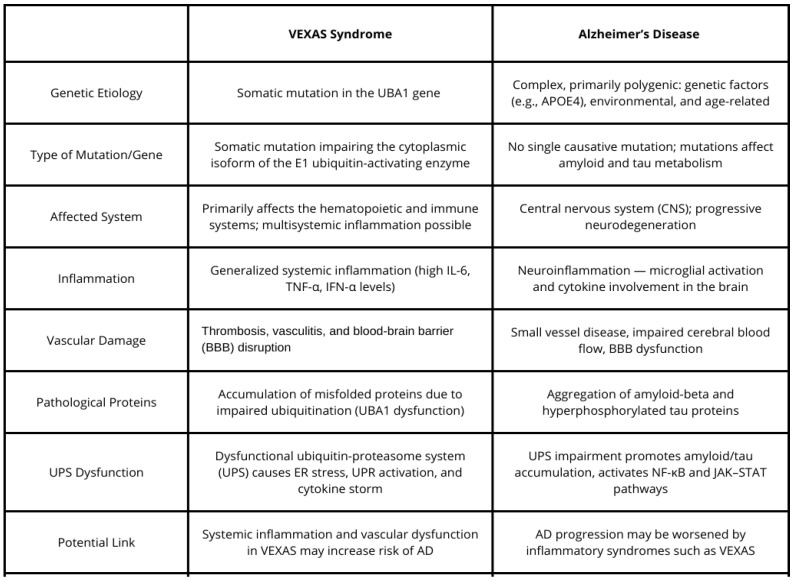
Comparative table summarizing key clinical, genetic, and molecular features of both conditions.

While VEXAS syndrome and Alzheimer’s disease are clinically distinct, they share overlapping pathophysiological features such as systemic inflammation, vascular dysfunction, and cellular stress mechanisms (Figure 5). Inflammation in VEXAS syndrome could potentially enhance neuroinflammation and contribute to the development of Alzheimer’s-like neurodegeneration. Moreover, vascular complications in VEXAS may exacerbate cognitive dysfunction through compromised blood–brain barrier integrity and impaired cerebral blood flow. The accumulation of misfolded proteins and aging-related immune dysfunction further links the two conditions at a molecular level. Given the similarities between VEXAS syndrome and Alzheimer’s disease, we propose that VEXAS may potentially increase the risk of developing AD. This hypothesis warrants further investigation to clarify the nature of the possible relationship between these two conditions and to deepen our understanding of their individual pathologies. Exploring the shared mechanisms may not only enhance our insight into each disease but also uncover new avenues for therapeutic intervention. Moreover, patients affected by one—or possibly both—of these conditions could benefit from such research, as treatments effective in one disorder might prove beneficial in the other due to their overlapping pathological features.

## Data Availability

No new data were created or analyzed in this study. Data sharing is not applicable to this article.
